# Elevated expression of placental growth factor is associated with airway-wall vascular remodelling and thickening in smokers with asthma

**DOI:** 10.1038/srep43017

**Published:** 2017-02-21

**Authors:** Dong Wu, Tianwen Lai, Yalian Yuan, Min Chen, Jun Xia, Wen Li, Guihai Pan, Binfan Yuan, Quanchao Lv, Yanyu Li, Dongmin Li, Bin Wu

**Affiliations:** 1 Institute of Respiratory Diseases, Department of Respiratory, The Affiliated Hospital of Guangdong Medical University, Zhanjiang 524001, China; 2 Department of Radiology, The Affiliated Hospital of Guangdong Medical University, Zhanjiang 524001, China

## Abstract

The increased expression of placental growth factor (PlGF) in chronic obstructive pulmonary disease and allergy-related asthma suggests its role in the pathogenesis of these diseases. In asthmatic smokers, airway remodelling is accompanied by an accelerated decline in lung function. However, whether PlGF contributes to the persistent airflow obstruction and vascular remodelling typically seen in asthmatic smokers is unknown. In this study we measured lung function, airway-wall thickening, and PlGF levels in serum and induced sputum in 74 asthmatic and 42 healthy smokers and never-smokers. Using human lung microvascular endothelial cells (HLMECs), we evaluated the *in vitro* effects of PlGF on each step of vascular remodelling, including proliferation, migration, stress-fibre expression, and tubule formation. Our data showed significantly higher serum and sputum PlGF levels in asthma patients, especially asthmatic smokers, than in healthy controls. Serum and sputum PlGF levels correlated negatively with post-bronchodilator forced expiratory volume in 1 s (FEV_1_) and the FEV_1_/forced vital capacity, but positively with airway-wall thickening. Stimulation of HLMECs with rhPlGF promoted all of the steps of airway-microvascular remodelling. These findings provide insights into the influence of cigarette smoking on the structural changes in the airways of asthmatics and the important pathogenic role played by PlGF.

In China, the prevalence of smoking in the asthmatic population is as high as 34.51%^1^. These individuals suffer from respiratory symptoms and a reduction in lung function parameters but are resistant to corticosteroid treatment^2^,^3^. Airway abnormalities, as seen on high-resolution computed tomography (HRCT), are worse in smoking than in never-smoking asthmatics^4^. In the former, sputum levels of proangiogenic factors are higher, which probably reflects ongoing angiogenesis in the airways and may serve as a biomarker of disease severity^5^,^6^.

The airways of smokers with asthma are characterized by an increase in the number and size of vascular structures. These alterations are mediated by angiogenic factors such as vascular endothelial growth factor (VEGF), osteopontin, and angiopoietin-1 and angiopoietin-2, all of which contribute to airway remodelling and airflow obstruction^5^,^6^,^7^,^8^. VEGF mediates the vascular and extravascular remodelling that contributes to the pathology of asthma^9^, by inducing vascular endothelial cell proliferation and tubule formation and by increasing microvascular permeability^10^. Microvascular endothelial cells have a crucial role in growth factor-induced angiogenesis based on their ability to migrate and form capillary-like structures and to recruit circulating immune cells, which aggregate at sites of inflammation^11^. Human lung microvascular endothelial cells (HLMECs) are actively involved in the pathogenesis of airway-wall vascular remodelling in asthmatic patients^12^. The formation of new blood vessels is supported by placental growth factor (PlGF), which acts directly on existing endothelial cells^12^,^13^.

PlGF is a member of the VEGF family, which includes VEGF-A, B, C, D, and E^14^. It is involved in pathological angiogenesis but is dispensable for physiological angiogenesis^15^. The higher-level expression of PlGF and VEGF receptor 1 in the sputum of patients with asthma was reported^16^, as was the increased expression of PlGF in the serum and bronchoalveolar lavage fluid of patients with chronic obstructive pulmonary disease (COPD)^17^. In the mouse lung, the over-expression of PlGF causes emphysema, by stimulating autophagy and apoptosis in type II pneumocytes^18^,^19^, whereas knocking-out PlGF protects mice against elastase-induced pulmonary emphysema^20^. We previously showed that cigarette-smoke extract (CSE) directly induces PlGF mRNA and protein expression as well PlGF secretion from bronchial epithelial cells in a dose- and time-dependent manner and that this sequence of events is mediated by the ROS/MAPK (ERK1/2)/Egr-1 pathway [our another paralleled study, unpublished data]. These observations suggest a key role for PlGF in the inflammation and neovascularization seen in smoking-related chronic inflammatory diseases of the airways. However, neither the expression pattern of PlGF nor the mechanism by which this growth factor activates angiogenesis during airway remodelling in asthma patients who are also smokers has been investigated.

The work described herein was a part of a study on biomarkers of COPD and asthma^21^,^22^,^23^. We hypothesized that the up-regulation of PlGF expression would be more pronounced in asthmatic patients who were smokers than in never-smokers with asthma and that higher PlGF levels played a functional role in airway remodelling, might throug promoting angiogenesis. Both hypotheses were tested by measuring lung function, examining airway-wall thickness by HRCT, and determining the PlGF levels in the serum and induced sputum of 74 asthmatics and 42 healthy smokers and never-smokers. In addition, in an *in vitro* study, we evaluated the effects of recombinant human PlGF (rhPlGF) on the proliferation, migration, stress-fibre expression, and tubule-formation of primary HLMECs.

## Results

### Demographics and clinical characteristics

The 74 asthmatics (30 smokers and 44 never-smokers) and 42 healthy individuals (20 smokers and 22 never-smokers) who participated in the study were matched for age, sex, and smoking history. Their demographic characteristics are summarized in Table 1. None of the participants were on oral corticosteroids therapy. The never-smokers with asthma were similar to the smokers with asthma in terms of age, duration of asthma, body surface area, dose of inhaled corticosteroid, total cell counts in induced sputum, and eosinophil and macrophage percentages, but the latter group had a higher neutrophil proportion. Sputum induction was well tolerated by all study participants.

### PlGF levels in the serum and induced sputumh

**Figure 1:**
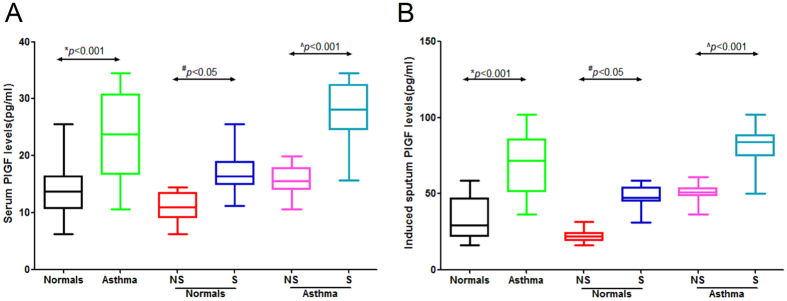
Serum and induced sputum placental growth factor (PlGF) levels in patients with asthma and in healthy controls. The PlGF levels in serum and induced sputum were assessed in patients with asthma [n = 74, never-smokers (NS), smokers (S)] and in healthy controls [n = 42, never-smokers (NS), smokers (S)] using an ELISA. (**A**) Serum PlGF levels were increased in patients with asthma compared with the healthy controls (*p < 0.001). When patients were stratified according to cigarette-smoking status, serum PlGF levels in smokers with asthma were higher than those in never-smokers with asthma (^^^*p* < 0.05), and those in healthy smokers were higher than in healthy never-smokers (^#^*p* < 0.001). (**B**) Similar results were obtained for PlGF levels in the induced sputum. *p < 0.001 for asthma, ^^^*p* < 0.001 for smokers with asthma, ^#^*p* < 0.05 for healthy smokers. Values are presented as the median (interquartile range).

Serum PlGF levels were significantly higher in patients with asthma than in the healthy controls. Smoking significantly increased the serum PlGF levels in smokers with asthma [median, interquartile ranges 27.1 (18.6–38.5)] vs. never-smokers with asthma [15.8 (9.6–19.9)] and in healthy smokers [16.3 (10.3–24.8)] vs. healthy never-smokers [10.8 (6.3–14.9)] (Fig. 1A). Similar results were obtained for PlGF levels in induced sputum [83.6 (39.1–102.3) vs. 48.9 (34.6–66.9), 45.7 (30.9–59.3) vs. 23.9 (16.3–38.9), respectively] (Fig. 1B). Multivariate regression analyses showed that age, sex, disease duration, body surface area, dose of inhaled corticosteroid, and sputum cell count and percentage were not significantly related to the PlGF level, whereas smoking (pack-years) had a significant effect on the PlGF levels in serum [r = 0.286 (95% CI 3.2–11), p = 0.001] and induced sputum [r = 0.435 (95% CI 7.6–44), p < 0.001] (Table 2).

### Correlation of serum and induced sputum PlGF levels with lung function

**Figure 2:**
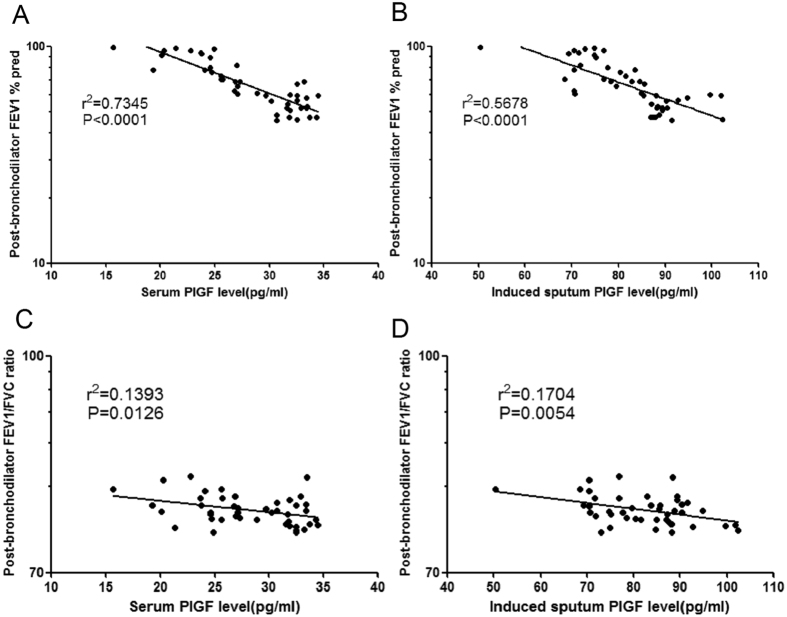
Association of serum and induced-sputum PlGF levels with lung function. (**A**,**B**) Serum and induced sputum PlGF levels correlated negatively with post-bronchodilator FEV1% pred [*r*^*2*^ = 0.7345*, P* < 0.0001 and *r*^*2*^ = 0.5678, *P* < 0.0001, respectively] and (**C**,**D**) post-bronchodilator FEV1/FVC ratio [*r*^*2*^ = 0.1393, *P* = 0.0126 and *r*^*2*^ = 0.1704, *P* = 0.0054, respectively].

Never-smokers with asthma were similar to smokers with asthma according to spirometry but they had a higher *D*_LCO_ (84.6% vs. 73.3% predicted, *p* = 0.003) and a higher *Fe*NO_50_ (24.3 ppb vs. 13.5 ppb, *p* < 0.001). As expected, healthy never-smokers had better lung function parameters than healthy smokers. Among smoking asthmatics, serum and induced sputum PlGF levels were negatively associated with post-bronchodilator FEV_1_% pred [*r*^*2*^ = 0.7345*, P* < 0.0001 and *r*^*2*^ = 0.5678, *P* < 0.0001, respectively] (Fig. 2A,B) and with post-bronchodilator FEV_1_/FVC ratio [*r*^*2*^ = 0.1393, *P* = 0.0126 and *r*^*2*^ = 0.1704, *P* = 0.0054, respectively] (Fig. 2C,D). There were no significant associations between PlGF levels and lung-function parameters, except for sputum PlGF levels and *D*_LCO_ in never-smokers with asthma [−0.356 (−0.635 to −0.138)] (Supplemental Table 1). None of the associations between PlGF levels and lung function parameters in healthy smokers and healthy never-smokers were significant (Supplemental Table 1).

### Correlation of serum and induced sputum PlGF levels with airway-wall dimensions

**Figure 3:**
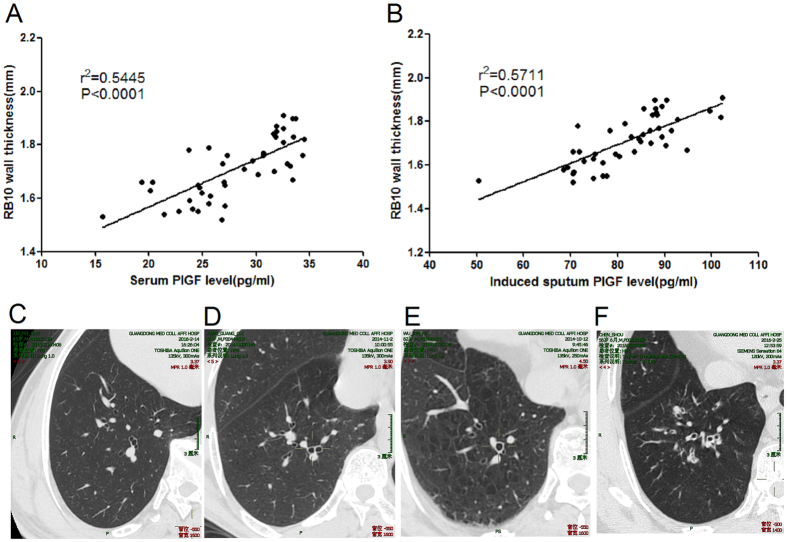
Association of serum and induced sputum PlGF levels with airway-wall thickening in smokers with asthma. (**A**,**B**) Serum and induced sputum PlGF levels correlated positively with right bronchial division 10 (RB10) airway-wall thickening in smokers with asthma [*r*^*2*^ = 0.5445, *P* < 0.0001, *r*^*2*^ = 0.5711, *P* < 0.0001, respectively]. (**C**) Representative images of high-resolution computed tomography of the airway wall. (**C**), Never-smoker healthy control; (**D**), healthy smoker; (**E**), never-smoker with asthma; (**F**), smoker with asthma.

Segmental airway-wall thickness, as seen on HRCT, was greater in smokers with asthma than in never-smokers with asthma [RB10 wall thickness: 1.83 mm (interquartile range, 1.62–2.12 mm) vs. 1.52 mm (1.21–1.70 mm) (*p* = 0.016)] and the airway lumen area was reduced [RB10 lumen area: 16.1 mm^2^ (interquartile range, 12.3–19.1 mm^2^) vs. 19.7 mm^2^ (14.5–24.3 mm^2^) (*p* = 0.011), respectively], but with no significant differences in RB10% wall area (Table 3). Chest HRCT showed positive correlations between serum and induced sputum PlGF levels and RB10 airway-wall thickness in smokers with asthma [*r*^*2*^ = 0.5445, *P* < 0.0001, *r*^*2*^ = 0.5711, *P* < 0.0001, respectively] (Fig. 3). Serum and induced sputum PlGF levels were not significantly associated with either RB10% wall area or RB10 lumen area in smoking asthmatics (Supplemental Table 2), nor were there significant associations between PlGF levels and RB10 CT measures in never-smokers with asthma (Supplemental Table 2).

### PlGF stimulates proliferation, migration, stress-fibre expression, and tubule formation in HLMECs

**Figure 4:**
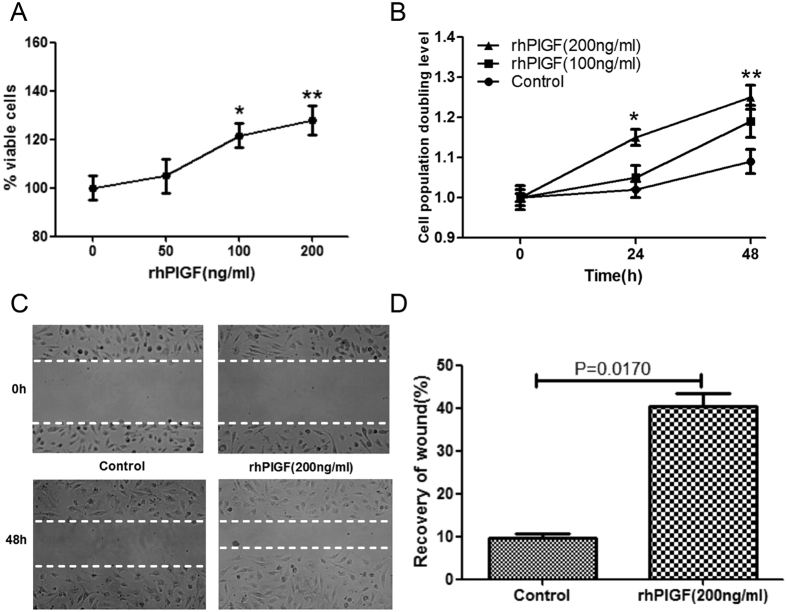
The effect of rhPlGF treatment on the proliferation and migration of human lung microvascular endothelial cells (HLMECs). (**A**) Cells treated with 0, 50, 100, or 200 ng rhPlGF/ml for 48 h and then resuspended and counted; (**B**) Cells treated with 0, 100, or 200 ng rhPlGF/ml for 0, 24, and 48 h, after which cell proliferation was measured using a CCK-8 assay. The number of rhPlGF -treated HLMECs was significantly higher than that of untreated control cells. (**C**) HLMECs at time 0 and after 48 h of incubation in the presence or absence of rhPlGF. Representative images of increased HLMEC migration in response to rhPlGF vs. untreated control cells. (**D**) The percentage of recovered wound area. The data are expressed as the mean ± standard error of the mean (SEM; n = 4 per group) of three independent experiments. *p < 0.05, **p < 0.01 vs. the basal level.

**Figure 5:**
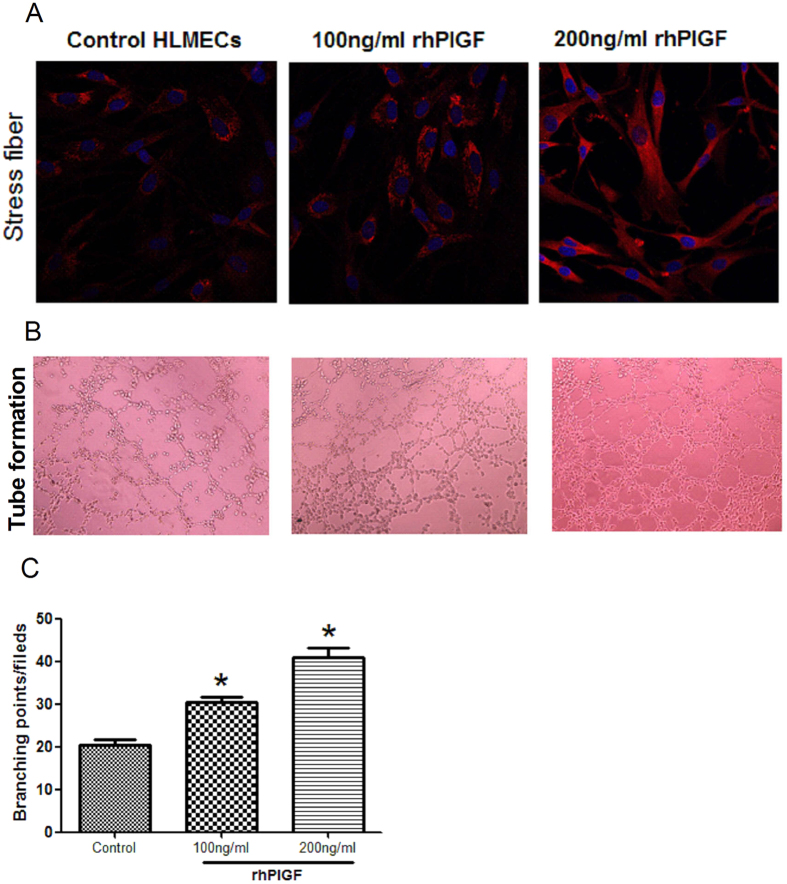
PlGF promotes stress-fibre expression and tubule formation in HLMECs. (**A**) HLMECs treated with 0, 100, or 200 ng rhPlGF/ml for 48 h. Stress-fibre expression after rhPlGF stimulation as determined by TRITC-phalloidin staining. (**B**) HLMECs (5 × 10^4^) treated or not with rhPIGF (100 or 200 ng/ml) were seeded onto 24-well plates containing Matrigel™ (10 mg/ml). The cells were incubated for 6 h to allow the formation of capillary-like structures on Matrigel and then imaged by phase-contrast microscopy (×40). (**C**) Quantitative data from the tube-formation assay. The results are expressed as the mean ± SEM of three independent experiments. *p < 0.05 vs. control HLMECs.

Based on the correlations of serum and induced sputum PlGF levels with RB10 airway-wall thickening in asthmatics who were smokers, we used HLMECs to investigate the proangiogenic effects of PlGF on the vascular remodelling of the airway wall. HLMECs are a well-established *in vitro* model of this process^12^. rhPlGF was added to the cells and its effects on several processes associated with angiogenesis were evaluated. The results showed that treatment with rhPlGF significantly increased both the number of primary HLMECs (Fig. 4A,B) and their migration capacity compared with the untreated control (Fig. 4C,D). Actin is a major cytoskeletal component of endothelial cells and the constant remodelling of the actin cytoskeleton into stress fibres is an essential step in angiogenesis^13^. TRITC-phalloidin staining, of HLMECs treated for 48 h with rhPlGF (100 and 200 ng/ml) showed that, compared with untreated control cells, the growth factor significantly increased stress-fibre formation in a concentration-dependent manner (Fig. 5A). The proangiogenic activity of PlGF in HLMECs was further examined by testing for *in vitro* tube formation using the Matrigel assay. Exposure of the cells to rhPlGF enhanced the formation of a capillary-like network, with the number of tubes significantly and dose-dependently increasing in response to rhPlGF addition (Fig. 5B,C).

## Discussion

The present study explored the potential role of PlGF in the relationship between cigarette smoking and asthma. Serum and sputum PlGF levels were significantly elevated in patients with asthma, especially in smokers vs. never-smokers. PlGF levels also correlated negatively with post-bronchodilator FEV_1_ and the FEV_1_/FVC ratio but positively with segmental airway-wall thickness in asthmatic patients who smoked. Stimulation of HLMECs with rhPlGF promoted proliferation, migration, stress-fibre expression, and tubule formation. Together, these results suggest an important role for PlGF in the pathobiologic characteristics seen in asthmatic smokers.

PlGF has been implicated in tissue ischemia, malignancy, inflammation, and several diseases^15^. In studies on its role in COPD, downstream signalling molecules were shown to contribute to the pathogenesis of airway epithelial cell apoptosis and emphysema^17^,^18^,^19^,^20^. PlGF has also been implicated in bronchial neutrophilic inflammation and oedema in allergic asthma^16^. Here we showed that sputum PlGF levels were significantly higher in patients with asthma than in non-asthmatics, consist with the findings of a previous study^16^. In addition, the serum and sputum PlGF levels of asthma patients were significantly higher in smokers than in never-smokers. This study is the first to evaluate the airway concentrations of PlGF in relation to the smoking status of patients with asthma. Multivariate regression analyses of this interaction failed to detect a relationship between age, sex, disease duration, body surface area, dose of inhaled corticosteroid, sputum cell count and percentage, and PlGF levels, whereas smoking (pack-years) was significantly related to PlGF levels in serum and induced sputum. The increased concentrations of PlGF in smoking asthmatics is in agreement with our previous finding of a direct dose- and time-dependent induction by CSE of PlGF mRNA and protein levels and of PlGF secretion from bronchial epithelial cells, via the ROS/MAPK (ERK1/2)/Egr-1 pathway (unpublished data). This observation is consistent with the results of this work, which demonstrated higher serum and sputum PlGF levels in healthy smokers than in healthy never-smokers. Together, these data provide evidence of the important effect of cigarette smoke on the physiological and pathological changes that characterize asthma, by augmenting PlGF expression.

A significant negative association between PlGF levels and post-bronchodilator FEV_1_ and FEV_1_/FVC ratio in smoking asthmatics was also detected in the present study. Cheng *et al*. similarly found an inverse correlation between PlGF levels and FEV_1_ in COPD patients^17^. The functional sequelae of airway remodelling in patients with asthma are believed to reflect the loss of a post-bronchodilator response to β^2^-adrenoceptor agonists^24^. In this context, our results suggest that the local generation of this proangiogenic factor induces airway remodelling in asthma patients who smoke. We showed that sputum PlGF correlated inversely with *D*_LCO_ in never-smokers with asthma [−0.356 (−0.635 to −0.138)], which indicates that the associated production of PlGF contributes to the compromised lung diffusion characteristic of these patients. However, the exact mechanism remains to be elucidated.

High-resolution CT has been used to quantify airway abnormalities arising from airway remodelling, and a correlation between the HRCT score and the severity of asthma as well as airflow obstruction has been reported^4^. In our asthma patients, HRCT abnormalities in RB10 airway wall thickness and lumen area were more prominent in smokers than in never-smokers; among the former, serum and induced sputum PlGF levels were positively associated with RB10 airway wall thickness and, in turn, the development of a severe expiratory flow limitation. In this process, PlGF, by promoting angiogenesis, may play a crucial role in dynamic airway remodelling.

Airway-wall neovascularization, manifested as increases in the number and size of bronchial blood vessels and angiogenic sprouts, is a prominent feature seen in asthma patients who are smokers and it correlates with airway-wall thickness, airway obstruction, and the degree of airway hyper-responsiveness^12^,^25^. Angiogenesis, a complex process whereby blood vessels sprout from the extant microvasculature, involves the sequential, coordinated degradation of the basement membrane by proteases, the proliferation and migration of endothelial cells, lumen formation, basement membrane reassembly, the recruitment of pericytes and/or vascular smooth muscle cells, vascular maturation, and, finally, blood flow^26^,^27^. We therefore postulated that an increased PlGF level contributes to the airway remodelling seen in smokers with asthma, might throug promoting angiogenesis in the airway wall. The role of PlGF in airway wall vascular remodelling in experiments was then further explored using HLMECs. Treatment with rhPlGF was shown to increase proliferation, migration, stress-fibre formation, and tubule formation by these cells, which in smokers with asthma may enhance the progression of small-airway remodelling. Our results are in line with those of an earlier report showing increased levels of PlGF in inflammatory bowel disease and the significant induction of migration and tubule formation by rhPlGF-1 in human intestinal microvascular endothelial cells^28^. Since the concentrations of rhPlGF used in our experiments were much higher than the highest levels of PlGF in the sputum. Therefore, our *ex-vivo* cell studies can only suggest a potential mechanism and further studies are needed to corroborate the findings and extend them.

There were several limitations to this study, including the absence of bronchial biopsies to confirm the serum, sputum, and CT findings. The cross-sectional design of the study design precluded the collection of data on longitudinal changes in PlGF levels in serum and sputum. Patients with asthma were not further assigned to sub-groups, as it would have reduced the statistical validity of the study. Also, HLMECs used in our experiments were not further purified to obtain bronchial arterial endothelial cells. Nonetheless, our results provide useful pilot data for larger studies of the effects of PlGF on asthmatic smokers.

In summary, our study revealed increases in the PlGF levels of the serum and induced sputum of asthmatic patients who are cigarette smokers. In this group, PlGF levels correlated positively with segmental airway-wall thickening and inversely with lung function. Our findings suggest that, in asthmatic smokers, PlGF contributes airway remodelling might through promoting angiogenesis. As such, they provide new insights into the pathophysiologic role of PlGF.

## Methods

### Subjects

The 74 asthmatic patients (current smokers and never-smokers) were recruited between October 2014 and February 2016 from respiratory clinics in the Affiliated Hospital of Guangdong Medical University, China. As controls, 42 healthy volunteers (healthy smokers and never-smokers) were recruited from the surrounding communities. All participants were on stable medication. Patients with a respiratory tract infection or an exacerbation of their asthma in the past 8 weeks were excluded. Smokers were defined as individuals with a smoking habit of at least 10 pack-years who currently smoke five or more cigarettes per day. Asthma was diagnosed according to the Global Initiative for Asthma (GINA) guidelines, based on a patient history of recurrent episodes of wheezing and chest tightness, with or without cough, impaired spirometry with reversibility, and a FEV_1_ of >12% and 200 ml after salbutamol administration or hyper-responsiveness to inhaled methacholine^29^. The Research Ethics Committee of the Affiliated Hospital of Guangdong Medical University approved the study, which was performed in accordance with the Declaration of Helsinki. All patients provided written informed consent.

This open cohort cross-sectional study of patients with asthma and healthy controls (smokers or never-smokers) was performed as part of a study on biomarkers of chronic inflammatory airway disease^21^,^22^,^23^.

### Sputum induction

Sputum was induced as previously described^5^ and using all modifications allowing safe measurement according to the severity of the underlying asthma. The sputum sample was divided in half, with one portion immediately treated with 10% dithiothreitol (DTT, Sigma Chemical, St Louis, MO, USA) and centrifuged at 400 g for 10 min, for inflammatory cell identification and the other diluted with phosphate-buffered saline (PBS) without DTT, for PlGF measurements. Both sputum supernatants were kept at −70 °C until used in further experiments.

### Enzyme-linked immunosorbent assay (ELISA)

Serum samples collected from patients with asthma and from healthy controls were kept at −70 °C. PlGF levels in serum and induced sputum were assessed using ELISA kits for human PlGF (R&D Systems, Minneapolis, USA) according to the manufacturer’s protocol.

### Pulmonary function tests

Spirometry was measured using standard spirometric techniques (Viasys Healthcare, Jaeger, Hoechberg, Germany) at least 6 h after the patient’s most recent treatment with albuterol, according to the guidelines of the American Thoracic Society (ATS)^30^. The fraction of exhaled nitric oxide was measured (Niox Flex; Aerocrine, Solna, Sweden) at 50 mL/s (FeNO_50_) in concordance with standardized guidelines^31^. Lung volumes and the lung diffusion capacity of carbon monoxide (*D*_LCO_) were determined according to ATS guidelines (Zan 500 body plethysmography, nSpire Health Limited, Hertford, UK)^32^.

### Chest computed tomography

CT of the chest was performed at full inspiration using a spiral CT scanner with 16-slice Brightspeed and 64-slice Lightspeed (GE Healthcare, Milwaukee, WI, USA) imaging and the following parameters: 125 kV, 100 mA·s, matrix size of 512 × 512, and a slice thickness of 1 mm. The window level was −600 Hounsfield units (HU), with a width of 1600 HU, as generally recommended for the evaluation of the bronchi and lung parenchyma^33^. Airway dimensions were measured by two observers (Prof Jun Xia and Dr Guihai Pan) blinded to the patient and control assignments. The software Pulmonary Work station 2.0 (VIDA Diagnostics, Iowa City, IA, USA) was used. The following CT airway values were obtained from the posterior basal segmental bronchus (designated as RB10) of the right lower lobe: wall area (%), wall thickness (mm), and lumen area (mm^2^). The measurements were performed once. Interobserver reproducibility was good (k = 0.7).

### HLMECs isolation and culture

Primary human HLMECs were isolated from lung tissue obtained from donors undergoing resection for localized lung carcinoma, as described previously^22^. All donors provided informed consent. The cells were re-suspended in endothelial cell medium (ECM, ScienCell, Carlsbad, CA, USA), seeded in fibronectin-coated (BD, Franklin Lakes, New Jersey, USA) flasks, and cultured at 37 °C in a 5% CO_2_ incubator. The culture medium was replaced every 2 days until the cells were confluent. All experiments were carried out using HLMECs between passages 5 and 8.

### Cell proliferation and migration assays

The cell proliferation assay was carried out in a 96-well plate using cells seeded at a density of 1 × 10^4^/well and treated for 48 h with different doses of recombinant human PlGF (rhPlGF, R&D Systems, Minneapolis, USA). A CCK-8 assay (CCK-8, Dojindo, Japan) to determine cell proliferation was performed according to the manufacturer’s instructions. HLMEC migration was assessed in a ‘scratch-wound’ assay^22^.

### Fibrillar actin staining and confocal microscopy

The structure of fibrillary actin (F-actin) was determined by TRITC-phalloidin (Sigma Chemical, St Louis, MO, USA) staining using HLMECs seeded on LabTek-II chamber slides (Fisher Scientific, Pittsburgh, PA, USA) at a density of 5 × 10^4^. The cells were cultured overnight and then stimulated for 1 h with different concentrations of rhPlGF (R&D Systems, Minneapolis, USA). They were then washed with PBS, fixed with 4% paraformaldehyde for 10 min, and permeabilized for 5 min with 0.5% Triton X-100 (Sigma Chemical, St Louis, MO, USA). Staining with TRITC-phalloidin (100 nM) for 30 min in the dark was followed by counterstaining for DNA with 4′, 6-diamidino-2-phenylindole (DAPI, Sigma Chemical, St Louis, MO, USA). F-actin was detected with a Leica-TCS-SP confocal microscope and the accompanying software (Leica v2.6.1).

### *In vitro* tubule formation

The formation of vascular-like structures by HLMECs on growth-factor-reduced Matrigel (BD Biosciences) was induced as previously described^34^. Briefly, Matrigel (250 μl) was added to the wells of a 24-well plate and allowed to polymerize for 30 min at 37 °C. HLMECs treated with vehicle or rhPlGF (100, or 200 ng/ml) were then layered on top of the Matrigel. After incubation of the cells for 6 h, tubule formation was assessed by inverted phase-contrast microscopy (Leica, Wetzlar, Germany). The number of branch points was counted from five randomly chosen microscopy fields (×40). Only points generating at least three tubules were counted.

### Statistical analysis

Normally distributed data are presented as the mean ± standard deviation (SD), and skewed data as the median (interquartile ranges). The Kolmogorov–Smirnov test was performed to examine the normality of the distribution. Statistical comparisons between groups were performed using a one-way analysis of variance (ANOVA) for normally distributed data and a Kruskal–Wallis test for skewed data, with the appropriate post-hoc tests performed for multiple comparisons (Bonferroni and Dunn’s, respectively). Differences in numerical variables between two groups were evaluated using unpaired t-tests or Mann–Whitney U-tests for normal and skewed data, respectively. Proportions were compared using χ^2^ tests. Categorical variables were summarized as the frequencies and percentages per category and compared using Fisher’s exact test or Pearson’s χ^2^ test. Associations between serum, induced sputum PlGF levels, and sputum cells were examined in a multivariate linear regression analysis in which either serum or the induced sputum PlGF level was the dependent variable. Linear regression analysis was performed with the covariates smoking, age, sex, body surface area, disease duration, and treatment regimen. A similar regression analysis was performed in two subgroups identified on the basis of smoking habit. The data were interpreted as standardized coefficients with 95% confidence intervals. Spearman’s or Pearson’s correlations were performed, according to data normality, between serum or induced sputum PlGF levels and lung function or chest CT parameters. SPSS 16.0 (Chicago, IL, USA) and GraphPad Prism 5.0 software (GraphPad Software Inc., San Diego, CA, USA) was used for the analyses and graphs. Statistical significance was set at *p* < 0.05.

## Additional Information

**How to cite this article:** Wu, D. *et al*. Elevated expression of placental growth factor is associated with airway-wall vascular remodelling and thickening in smokers with asthma. *Sci. Rep.* **7**, 43017; doi: 10.1038/srep43017 (2017).

**Publisher's note:** Springer Nature remains neutral with regard to jurisdictional claims in published maps and institutional affiliations.

## Supplementary Material

Supplemental Tables

## Figures and Tables

**Table 1:** Demographics and clinical characteristics of the study participants.

	Asthmatic patients		Normal controls	
	Never-smoker	Smoker	Never-smoker	Smoker
Subjects (n)	30	44	22	20
Age years	48.3 (42.0–53.0)	49.1 (41.0–54.0)	48.2 (40.0–55.0)	51.5 (44.0–58.0)
Male sex n (%)	14 (46.7)	20 (45.5)	10 (45.5)	9 (45.0)
Disease duration years	19.0 (8.5–31.0)	19.5 (10.5–33.5)		
Body surface area (m^2^)	1.92 (1.73–2.28)	1.83 (1.65–2.12)	1.79 (1.63–1.98)	1.90 (1.62–2.22)
Smoking pack-years		34.5 (24.0–61.0)		31.0 (23.0–58.0)
Beclometasone-equivalent dose of inhaled steroid (μg)	800 (450–1000)	800 (700–1050)		
Sputum total cell count x10^6^·mL^−1^	6.04 (5.25–8.47)	5.89 (4.88–7.61)	6.51 (5.68–7.88)	5.67 (5.07–6.21)
Sputum neutrophils (%)	16.8 (14.9–19.5)	23.0 (20.0–26.2)^#^	17.2 (12.2–21.0)	21.8 (16.6–24.8)*
Sputum eosinophils (%)	7.6 (3.3–11.1)	6.4 (2.3–10.4)	0.00 (0.00–0.29)	0.00 (0.00–0.22)
Sputum macrophages (%)	66.8 (61.6–74.9)	64.0 (59.5–70.5)	78.0 (75.6–84.5)	74.2 (70.4–78.1)
Lymphocytes (%)	1.8 (0.8–2.4)	1.4 (0.9–2.0)	1.1 (0.3–1.6)	0.9 (0.2–1.3)
Epithelial cells (%)	7.0 (4.9–8.8)	5.2 (3.2–6.8)	3.7 (0.9–6.1)	3.1 (1.4–5.6)

The data are presented as the median (interquartile range) or n (%), unless otherwise stated. ^#^Never-smokers with asthma vs. smokers with asthma *p* < 0.05; *healthy never-smokers vs. healthy smokers, *p* < 0.05.

**Table 2:** Multivariate regression analysis (as a single model) between serum, induced sputum PlGF levels, and clinical parameters in the whole study group.

Variables	Serum PlGF level			Induced sputum PlGF level		
	β standardized coefficient (95% CI)	Adjusted R^2^	*P* value	β standardized coefficient (95% CI)	Adjusted R^2^	*P* value
Age	0.08 (−0.17, 0.33)	0.002	0.484	−0.03 (−0.21, 0.12)	0.02	0.831
Gender	0.17 (−17, 8)	0.011	0.218	0.12 (−1.12, 1.43)	0.03	0.776
Duration of the Disease	−0.09 (−14, 4)	0.003	0.384	0.004 (−53, 61)	0.0005	0.954
Body surface area	0.14 (−1, 5)	0.006	0.183	0.108 (−1.8, 12)	0.008	0.151
Smoking	0.289 (3.2, 11)	0.286	0.001^*^	0.551 (7.6, 44)	0.435	<0.001^#^
Inhaled steroid treatment	−0.05 (−44, 28)	0.003	0.608	−0.064 (−12, 4)	0.005	0.241
Eosinophils%	0.18 (−18, 7)	0.013	0.209	−0.07 (−269, 209)	0.0004	0.823
Neutrophils%	−0.183 (68, 5)	−0.801	0.076	−0.433 (−13, 0.2)	0.093	0.061
macrophages%	0.163 (−34, 459)	0.091	0.112	0.118 (161, 209)	0.003	0.126

^*^Significant associations for serum PlGF level. ^#^Significant associations for induced sputum PlGF level. The covariates for the regression analysis were: age, sex, disease duration, smoking, body surface area, inhaled steroid treatment, and induced sputum cell proportions.

**Table 3:** Lung-function parameters and computed tomography measures of airway dimensions in patients with asthma and in healthy controls.

	Asthma			Healthy control		
	Never-smoker	Smoker	P value^#^	Never-smoker	Smoker	P value*
Lung function						
Pre-bronchodilator FEV1% pred	81.5 (72.5–93.0)	78.5 (58.5–91.5)	0.333	105 (98.8–113.2)	92.5 (87.5–99.5)	0.003*
Post-bronchodilator FEV1% pred	89.2 (81.3–98.1)	83.2 (76.4–99.6)	0.251			
Post-bronchodilator FEV1/FVC (%)	80.3 (77.4–84.6)	76.7 (75.3–83.4)	0.304			
*D*_LCO_% pred COHb	84.6 (80.3–93.1)	73.3 (64.2–85.1)	0.003^#^			
*Fe*NO_50_ ppb	24.3 (12.1–43.4)	13.5 (9.6–15.6)	<0.001^#^	12.3 (8.1–14.5)	6.8 (5.3–12.8)	0.036*
Computed tomography						
RB10% wall area mm^2^	60.3 (57.6–66.3)	64.1 (60.3–67.9)	0.083			
RB10 wall thickness mm	1.52 (1.21–1.70)	1.83 (1.62–2.12)	0.016^#^			
RB10 lumen area mm^2^	19.7 (14.5–24.3)	16.1 (12.3–19.1)	0.011^#^			

The data are presented as the median (interquartile range) or n (%), unless otherwise stated. ^#^Never-smokers with asthma vs. smokers with asthma; *Healthy never-smokers vs. healthy smokers. FEV_1_: forced expiratory volume in 1 s; FVC: forced vital capacity; D_LCO_% pred COHb: lung diffusion capacity of carbon monoxide corrected for haemoglobin and carboxyhaemoglobin, as a percentage of the predicted value; FeNO_50_: exhaled nitric oxide fraction measured at a flow rate of 50 mL · s^−1^; RB10: right bronchial division 10.
