# Supplementary material for: Elevated expression of placental growth factor is associated with airway-wall vascular remodelling and thickening in smokers with asthma
Source: Sci Rep. 2017 Feb 21;7:43017. doi: 10.1038/srep43017 (PMC5318961; doi:10.1038/srep43017)
Supplement: Supplemental Tables [file srep43017-s1.pdf]

Elevated expression of placental growth factor is associated with airway-wall vascular remodelling and thickening in smokers with asthma

Dong Wu[1,#], Tianwen Lai[1,#], Yalian Yuan[1,#], Min Chen[1], Jun Xia[2], Wen Li[1], Guihai Pan[2], Binfan Yuan[1], Quanchao Lv[1], Yanyu Li[1], Dongmin Li[1,*], Bin Wu[1,*]

[1] Institute of Respiratory Diseases, Department of Respiratory, The Affiliated Hospital of Guangdong Medical University, Zhanjiang 524001, China

[2] Department of Radiology, The Affiliated Hospital of Guangdong Medical University, Zhanjiang 524001, China

*To whom correspondence should be addressed:

Prof Bin Wu

Institute of Respiratory, The Affiliated Hospital of Guangdong Medical University, Zhanjiang 524001, China Tel: +86-07592386792 E-mail: wubin1190@126.com

Prof Dongmin Li

Department of Respiratory, The Affiliated Hospital of Guangdong Medical University, Zhanjiang 524001, China Tel: +86-07592386792 E-mail: 3064264@qq.com

[#] These authors contributed equally to this work.

Supplemental Table 1. Association between serum, induced sputum PlGF levels and lung function in four groups.

	Groups	Pre-bronchodilator FEV$_1$% pred	Post-bronchodilator FEV1 % pred	Post-bronchodilator FEV1/FVC	DLCO% pred COHb	FeNO$_{50}$
**serum PlGF levels**	Smoking asthmatics	0.162 (-0.184–0.494)	-0.735 (0.065–0.599)*	-0.139 (0.070–0.626)*	0.089 (-0.279–0.463)	0.168 (-0.132–0.435)
	Non-smoking asthmatics	0.217 (-0.135–0.563)	0.231 (-0.081–0.534)	0.181 (-0.134–0.465)	-0.098 (-0.398–0.229)	0.179 (-0.159–0.439)
	Smoking healthy control	0.284 (-0.089–0.545)				-0.228 (-0.591–0.124)
	Non-smoking healthy control	0.201 (-0.103–0.567)				-0.255 (-0.567–0.421)

**induced sputum PlGF levels**	Smoking asthmatics	0.225 (-0.064–0.515)	—0.568 (0.008-0.587)#	—0.170 (0.105-0.614)#	0.189 (-0.159–0.503)	-0.172 (-0.517–0.209)
	Non-smoking asthmatics	0.151 (-0.179–0.451)	0.173 (-0.159–0.465)	0.113 (-0.184–0.413)	-0.356 (-0.635– -0.138) #	0.065 (-0.284–0.367)
	Smoking healthy control	0.131 (-0.169–0.434)				-0.041 (-0.357–0.279)
	Non-smoking healthy control	0.103 (-0.249–0.425)				0.185 (-0.159–0.506)

*Indicate significant associations for serum PlGF level. #Indicate significant associations for induced sputum PlGF level. FEV1: forced expiratory volume in 1s; FVC: forced vital capacity; D_{LCO}% pred COHb: diffusing capacity of the lung for carbonmonoxide corrected for haemoglobin and carboxyhaemoglobin, as a percentage of the predicted value; $FeNO_{50}$: exhaled nitric oxide fraction measured at a flow rate of 50 mL·s^{-1}.

Supplemental Table 2. Association between serum, induced sputum PlGF levels and computed tomography RB10 airway dimensions in asthmatics

	Groups	RB10 % wall area	RB10 wall thickness	RB10 lumen area
**serum PlGF levels**	Smoking asthmatics	0.069 (-0.254–0.383)	0.545 (0.102–0.668)*	-0.038 (-0.364–0.312)
	Non-smoking asthmatics	0.034 (-0.276–0.415)	0.081 (-0.263–0.387)	0.213 (-0.067–0.551)
**induced sputum PlGF levels**	Smoking asthmatics	0.076 (-0.317–0.420)	0.571 (0.189–0.728)[#]	0.169 (-0.153–0.438)
	Non-smoking asthmatics	0.081 (-0.243–0.444)	0.138 (-0.249–0.431)	0.096 (-0.253–0.432)

*Indicate significant associations for serum PlGF level. #Indicate significant associations for induced sputum PlGF level.; RB10: right bronchial division 10.
